# Different diseases, same circuits: lessons from rare and overlooked causes of disorders of consciousness

**DOI:** 10.1093/braincomms/fcaf439

**Published:** 2025-11-10

**Authors:** Daniel Toker, Martin M Monti

**Affiliations:** Department of Neurology, University of California, Los Angeles, CA 90095, USA; Department of Psychology, University of California, Los Angeles, CA 90095, USA; Department of Psychology, University of California, Los Angeles, CA 90095, USA; Brain Injury Research Center, Department of Neurosurgery, University of California, Los Angeles, CA 90095, USA

**Keywords:** consciousness, coma, disorders of consciousness, rare diseases, brain injury

## Abstract

Most foundational frameworks for understanding disorders of consciousness are based on common aetiologies such as traumatic brain injury, stroke or hypoxic–ischemic insult. From these, the mesocircuit hypothesis has emerged as a leading model, proposing that consciousness depends on a distributed network of brainstem arousal systems, central thalamic hubs and cortico–subcortical loops. However, rare and underrecognized causes of coma and prolonged disorders of consciousness offer a unique opportunity to test, refine and expand these models. In this review, we analyse a wide array of rare aetiologies—including genetic syndromes, parasitic and fungal infections, autoimmune encephalitides, amyloid and tau pathologies, toxic-metabolic states and haematological disorders—that are often excluded from mainstream consciousness research. Despite their diverse mechanisms, these conditions converge on a consistent set of anatomical targets: the central thalamus, brainstem reticular activating system, deep white matter ‘bridging zones’, basal ganglia and distributed cortical networks. This convergence not only provides powerful external validation of network-based frameworks such as the mesocircuit hypothesis but also underscores the clinical need for therapeutic approaches that address both aetiology-specific pathology and distributed circuit-level dysfunction. However, evidence quality varies considerably across these rare conditions, with many findings based on limited case reports or small series, necessitating cautious interpretation of proposed anatomical frameworks.

## Teaser

Toker and Monti report that rare causes of disorders of consciousness—including infections, autoimmune conditions, genetic disorders and neurodegenerative diseases—target the same brain circuits as common causes: brainstem arousal systems, central thalamus, basal ganglia and distributed cortical networks. Their review validates network-based frameworks for understanding arousal and awareness.

## Graphical Abstract

**Graphical Abstract:**
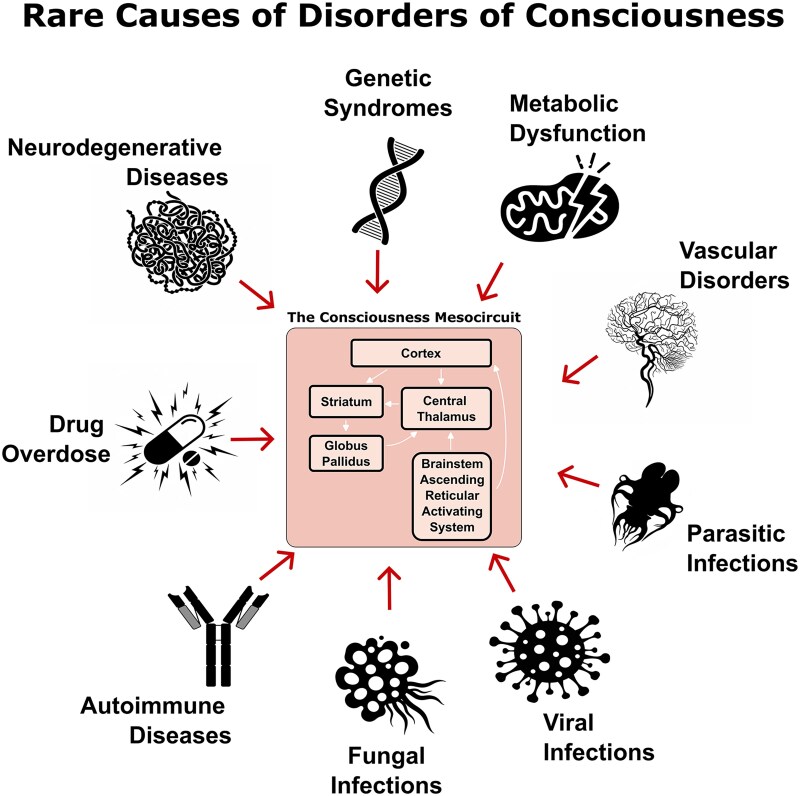

Impact statement Rare and overlooked causes of disorders of consciousness offer a natural stress test for existing models of consciousness. This review demonstrates that despite wide variation in their underlying mechanisms, such conditions consistently affect the same core circuits implicated in common causes of disorders of consciousness: brainstem arousal systems, central thalamic nuclei, basal ganglia, deep white matter ‘bridging zones’, and distributed cortical networks. These findings strengthen leading theoretical frameworks such as the mesocircuit hypothesis and highlight the inherently network-based nature of arousal and awareness. The convergence of anatomical targets across diverse pathological mechanisms provides compelling evidence of a recurring cross-aetiology pattern that transcends individual case limitations. Clinically, they reveal the need for dual therapeutic strategies that combine aetiology-specific interventions (e.g. antimicrobial, immunologic or metabolic therapies) with broader circuit-level approaches aimed at restoring connectivity and function across distributed systems.

## Introduction

Consciousness refers to the capacity for subjective experience, including the potential for awareness of self and environment, even in the absence of overt behavioural responsiveness. Disorders of consciousness (DOC), arising from severe brain injuries, disrupt these capacities and present profound clinical challenges for patients, families and healthcare professionals.

Broadly, the spectrum of DOC includes coma, which is characterized by profound disruption to both arousal and awareness; the vegetative state/unresponsive wakefulness syndrome (VS/UWS), which is characterized by wakefulness without awareness; the minimally conscious state (MCS), which is characterized by fluctuating but reproducible signs of awareness; and emergence from MCS, which is characterized by object use or functional communication.^1-8^ Importantly, these are clinical syndromes—defined by impaired arousal and/or awareness—rather than disease entities with well-defined lesion patterns.^9^ Moreover, the boundaries between these syndromes can be tenuous: coma is frequently a transitory phase in the broader trajectory of consciousness disorders, with many prolonged forms of DOC (defined as lasting ≥28 days^7^) originating in an initial episode of acute coma. Conversely, as we review below, some conditions can lead to a deterioration of the content of consciousness (awareness) without an early, primary compromise of arousal.

Over recent decades, our understanding of the neural mechanisms underlying the loss and recovery of consciousness after severe brain injury has significantly advanced, moving from reductionist models towards complex frameworks emphasizing the distributed networks that sustain wakeful awareness.^10^ Crucially, much of this progress has been informed by studies of common aetiologies, such as traumatic brain injury (TBI), stroke and anoxic brain injury, which collectively cause the majority of non-induced coma cases.^11^

Brain injuries offer natural lesion studies that dissociate critical components of consciousness, including arousal, awareness and behavioural responsiveness. These dissociations are uniquely observable in clinical conditions such as VS/UWS, where cyclical arousal persists despite the absence of detectable awareness.^12,13^ Widely adopted frameworks therefore conceptualize consciousness along two primary dimensions: the level of consciousness, encompassing wakefulness or arousal regulated predominantly by brainstem systems and their broad projections,^14^ and the content of consciousness, involving awareness dependent on integrated cortical–subcortical interactions.^10^

The foundational role of brainstem structures in consciousness emerged from the seminal early work by Bremer showing that selective pontomesencephalic brainstem lesions cause coma,^15^ and the work by Moruzzi and Magoun demonstrating that electrical stimulation of the ascending reticular activating system (ARAS) promoted wake-like EEG patterns while its ablation causes coma.^16^ More contemporary research now recognizes the ARAS as comprising multiple neurochemically distinct systems, including cholinergic, noradrenergic and serotonergic nuclei, each contributing specific arousal functions.^17-20^ Clinical studies have further refined localization of coma-producing lesions within the brainstem, pinpointing areas such as the upper pontine tegmentum and rostral dorsolateral pontine tegmentum as crucial sites whose disruption leads to profound impairments of consciousness.^21,22^

Beyond the brainstem, the thalamus acts as a pivotal integration hub for consciousness. The central thalamic nuclei, particularly the intralaminar and related paralaminar nuclei, integrate arousal signals with cortical attentional networks, facilitating distributed cortical dynamics essential for consciousness.^23,24^ Damage to these nuclei is consistently implicated in severe DOC, with post-mortem and *in vivo* studies revealing clear relationships between thalamic atrophy and clinical outcomes, behavioural estimates of depth of unconsciousness and EEG correlates of consciousness.^25-28^ The selective importance of the thalamus in coma is perhaps best illustrated by artery of Percheron (AOP) infarction, a rare anatomical variant of a frequent cause of coma (i.e. stroke). AOP occlusion produces bilateral paramedian thalamic infarcts precisely targeting dorsomedial and intralaminar nuclei, often resulting in coma.^29-35^ Though coma from AOP infarction may involve concurrent midbrain damage,^9^ imaging suggests patients can develop coma even when AOP infarction spares the midbrain.^31,35^

The content and quality of conscious experience is thought to depend critically on the interactions between these subcortical structures and broad cortical networks. Indeed, cortical networks traditionally associated with awareness and self-reflection—such as frontoparietal and default mode regions—often show metabolic disruption in VS/UWS, suggesting that impairments in higher-order associative cortex are common in DOC.^36-38^ However, subcortical structures, particularly the basal ganglia, also play a critical role. The striatum and globus pallidus contribute to cortico–subcortical loops that support cognitive, motor and limbic functions. Medium spiny neurons in the striatum are especially vulnerable to metabolic stress, which contributes to their frequent involvement in hypoxic–ischemic injury.^39,40^ Pallidal involvement is also apparent in DOC: atrophy in the globus pallidus, but not the thalamus, has been shown to correlate with reduced perturbational complexity index—a physiological marker of consciousness.^28^ Additionally, basal ganglia atrophy is associated with impairments in arousal.^27^ While the internal segment of the globus pallidus (GPi) has long been implicated in excessive thalamic inhibition, more recent studies suggest that the external segment of the globus pallidus (GPe) may play a key role in regulating arousal and electrocortical activity: tractographic evidence demonstrates direct GABAergic projections from GPe to the prefrontal cortex and central thalamus, particularly the central medial intralaminar nucleus, positioning the GPe as a key modulator of arousal-related circuits independent of GPi-mediated thalamic inhibition.^41^

**Figure 1:**
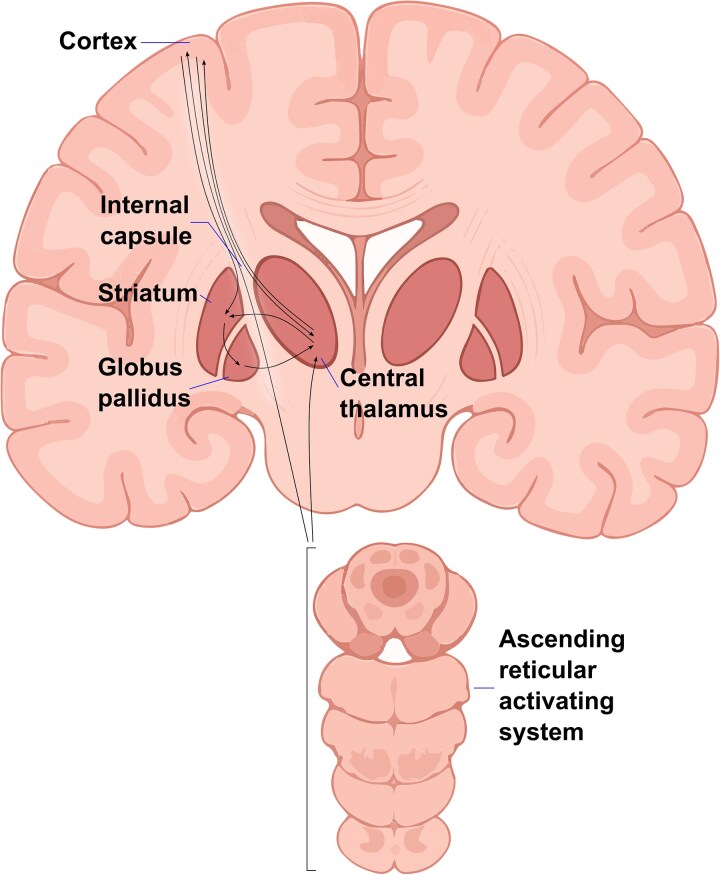
**The consciousness mesocircuit.** This diagram illustrates the critical anatomical substrates consistently targeted by both common and rare/overlooked causes of DOC. The circuit comprises the cortex, striatum, globus pallidus and central thalamic nuclei, connected by thalamocortical, corticothalamic and corticostriatal projections (black lines) that traverse the deep white matter bridging zone via the internal capsule. The ARAS in the brainstem provides the fundamental arousal substrate for consciousness and sends direct projections to the thalamus and to the cortex (black lines), with cortical projections also passing through the internal capsule. Rare aetiologies of acute coma and prolonged DOC demonstrate remarkable anatomical convergence on these specific neural circuits despite vastly different underlying pathophysiology, validating foundational frameworks for understanding the neural substrates of arousal and awareness.

Several of these insights have been synthesized in the mesocircuit hypothesis, proposed by Schiff, which describes a specific circuit comprising the cortex, striatum, globus pallidus and central thalamus (Fig. 1). According to this framework, severe brain injuries produce widespread deafferentation that maximally impacts central thalamic neurons due to their unique vulnerability—these neurons provide the shortest point-to-point connections across the entire corticothalamic system and thus suffer the greatest impact from multifocal disconnection.^42,43^ This primary central thalamic dysfunction causes a cascade of secondary effects: the loss of excitatory thalamic output reduces activity throughout frontal cortex and striatum, leading to shutdown of striatal medium spiny neurons, which have high firing thresholds and depend on robust corticostriatal and thalamostriatal input.^39^ The silencing of medium spiny neurons disinhibits the globus pallidus, which then actively inhibits the already disfacilitated central thalamus, creating a vicious cycle that further suppresses thalamocortical activity and impairs both arousal and awareness.^42,44^ This account represents both a localizationist framework—emphasizing specific anatomical nodes critical for consciousness—and a diaschitic one,^1,45^ as it describes how dysfunction cascades through interconnected circuit components to produce widespread functional depression.

Beyond fitting to observed patterns of injury in DOC, this model aims to explain pharmacological responses in DOC patients, such as improvements with dopaminergic medications, particularly amantadine,^46-48^ which potentiate striatal inhibition of the globus pallidus, or paradoxical responses to zolpidem,^48-50^ which inhibits the globus pallidus and is thereby thought to disinhibit thalamocortical networks. Indeed, PET imaging studies have confirmed that zolpidem response in DOC involves metabolic reactivation of prefrontal areas, corroborating the mesocircuit hypothesis and highlighting the key role of prefrontal cortex in recovery of functional communication.^51^ Further support comes from PET imaging studies of amantadine, which demonstrate significant increases in metabolic activity across bilateral dorsolateral prefrontal, temporoparietal and mesiofrontal cortices in MCS.^52^ These findings, alongside evidence from an expanding array of pharmacological interventions—including anticonvulsants like lamotrigine,^53^ other dopaminergic agents (levodopa,^54^ bromocriptine,^55^ apomorphine,^56-58^ selegiline^59^) and GABAergic modulators (baclofen^60-63^)—reinforce the view that consciousness recovery depends on restoring function across distributed mesocircuit components.

Importantly, however, these insights have largely emerged from studies of common DOC aetiologies (e.g. TBI, stroke, anoxia). Yet, a range of less common or overlooked conditions, from genetic disorders to dementia, can also result in DOC, and analysing the pathophysiology of these conditions presents a unique opportunity to further validate and refine our basic and clinical understanding of consciousness.

Here, we undertake a review of rare and/or overlooked aetiologies of acute coma and prolonged DOC, examining isolated case studies and series spanning diverse pathophysiological mechanisms, including metabolic disorders, neurodegenerative disorders, autoimmune conditions, infections and genetic syndromes. We organize our analysis by documented clinical presentations—cases presenting with acute coma versus those with prolonged DOC—while recognizing that this distinction is somewhat artificial. As we review below, many conditions manifest across this spectrum: some begin with acute coma and evolve through various DOC states towards recovery or chronic unconsciousness, while others (particularly progressive neurodegenerative conditions) lead to unresponsive states through gradual network failure without initial vigilance compromise. This organizational approach, however, allows us to examine what specific anatomical patterns emerge in different clinical contexts and trajectories, providing insights into both the acute disruption and progressive deterioration of consciousness networks.

By analysing these uncommon presentations, we aim to re-examine and contextualize current neural substrate models of consciousness—particularly the mesocircuit hypothesis—while identifying divergent trajectories or outlier presentations that may refine these theoretical frameworks. This dual approach allows us to consolidate evidence supporting existing circuit-based models while exploring how rare cases might expand or stress test those models, with particular emphasis on specific white matter tracts and the critical role of focal lesions in mesocircuit nodes.

It is, however, crucial to emphasize that the evidence quality across rare or generally underrecognized DOC aetiologies varies dramatically, reflecting the inherent challenges of studying uncommon conditions. Many findings rely on single case reports or small case series, limiting our ability to establish definitive causal relationships between specific anatomical lesions and consciousness impairment. While we emphasize anatomical convergence patterns across these diverse aetiologies, readers should note that the strength of evidence differs substantially between conditions with multiple documented cases versus those reported in isolated case reports. This heterogeneity in evidence quality necessitates cautious interpretation of the proposed anatomical frameworks and highlights the need for systematic multicentre studies and standardized consciousness assessment protocols in these rare conditions.

In addition, it is essential to also acknowledge that behavioural phenotyping of DOC itself is often confounded by factors unrelated to the core impairment of consciousness.^64^ Indeed, in the absence of standardized neurobehavioural assessment tools with high diagnostic sensitivity, such as the Coma Recovery Scale-Revised, misdiagnosis rates have been estimated to be as high as 40% of cases.^65-67^ Underlying central and peripheral impairments, such as aphasia, neuromuscular abnormalities and sensory deficits, may mask conscious awareness,^7^ while damage to motor systems frequently accompanying DOC means that patients might retain awareness but have insufficient voluntary control over their movements to demonstrate this through observable behaviours.^68^ Indeed, recent evidence indicates that approximately 25% of behaviourally unresponsive patients demonstrate cognitive motor dissociation (CMD)—an uncoupling between residual cognitive abilities and motor expression capacity detectable through functional neuroimaging or EEG.^69,70^ Specific conditions further complicate assessment: akinetic mutism presents with intact consciousness but profoundly diminished goal-directed behaviour,^71^ cerebellar mutism syndrome involves speech absence and hypotonia that can mimic consciousness impairment,^72^ and locked-in syndrome may be misdiagnosed as VS/UWS due to quadriplegia and anarthria despite preserved awareness.^9,73^ These differential diagnoses, along with the possibility of CMD, must be considered not only in common aetiologies like TBI and cerebral anoxia but also in the rare aetiologies reviewed below, as ignoring these confounders risks misclassification and mismanagement.^74^ The convergence patterns identified in rare DOC aetiologies must, therefore, be interpreted within this broader context of diagnostic complexity, emphasizing the need for comprehensive assessment protocols that account for both behavioural confounders and the potential for preserved cognitive function despite motor unresponsiveness.

## Rare and overlooked causes of acute coma

We first review rare causes of acute coma, which represents a fundamental disruption of consciousness networks. While common aetiologies such as TBI, stroke and anoxic brain injury have established the likely anatomical foundations of consciousness—particularly the ARAS, central thalamic nuclei and distributed cortical networks—rare causes provide unique opportunities to validate and refine these frameworks. As we show below, these uncommon conditions demonstrate remarkable anatomical convergence despite vastly different underlying pathophysiology, frequently targeting mesocircuit nodes or disrupting the white matter pathways connecting them (Table 1). This convergence across diverse pathological mechanisms provides compelling evidence that consciousness depends on specific network integrity rather than the nature of the disrupting process.

**Table 1:** A summary of rare and/or overlooked aetiologies with documented cases presenting with or progressing through acute coma

Aetiology	Nature of condition	Primary sites affected	Recovery trajectory	References
Wernicke's encephalopathy	Thiamine deficiency (metabolic)	Medial/dorsomedial thalamus, periaqueductal midbrain, striatum, cortex	Variable	^9,75-77^
Bickerstaff brainstem encephalitis	Autoimmune inflammation	Brainstem	Variable	^78-81^
Reye syndrome	Hyperammonemia (toxic-metabolic)	Thalamus, midbrain, basal ganglia, cortex, periventricular white matter	Variable	^82-84^
Heroin toxicity (including ‘chasing the dragon’)	Toxic-metabolic	Periventricular white matter, thalamus, internal capsule, globus pallidus	Variable	^85-88^
Multiple sclerosis (coma cases)	Autoimmune demyelination	Periventricular white matter, corpus callosum, pons	Complete recovery	^89^
Susac syndrome	Autoimmune microangiopathy	Corpus callosum, internal capsule, basal ganglia, brainstem, cortex	Variable	^90-93^
African trypanosomiasis	Parasitic infection	Internal capsule, corpus callosum, basal ganglia, cortex, brainstem, thalamus	Variable	^94-98^
Thrombotic thrombocytopenic purpura	Microangiopathic blood disorder	Periventricular white matter, cortex (functional hypoperfusion), pons	Variable	^99,100^
Primary CNS vasculitis	Inflammatory vascular disease	Periventricular white matter, cortex, brainstem	Poor prognosis, fatal in most cases	^101-103^
MELAS	Mitochondrial cytopathy	Cortex, periventricular white matter, midbrain	Transient, recovery with treatment	^104-106^
Hypoglycaemia	Metabolic	Cortex, internal capsule, periventricular white matter	Early recovery or irreversible unconsciousness	^107-114^
Fungal infections (e.g. *Cladophialophora*, *Aspergillus*)	Opportunistic infection	Brainstem (pons, midbrain), corona radiata, periventricular white matter	Poor prognosis, typically fatal	^115-120^
Tricyclic antidepressant overdose	Toxic (pharmacologic)	Functional brainstem suppression (no structural lesions)	Complete recovery with drug clearance	^121-127^
Acute intermittent porphyria	Metabolic (heme synthesis defect)	Bilateral cortical lesions (frontal, parietal, occipital)	Transient, recovery typical	^128^
Cerebral venous sinus thrombosis	Vascular occlusion (venous)	Posterior cortex (oedema), diffuse cortical congestion	Good recovery in most cases	^129^
Parkinson's-associated episodes	Neurodegeneration + neurotransmitter depletion	Basal ganglia (striatum)	Transient, complete recovery	^130-133^

In what follows, as an organizational principle, we present these rare aetiologies in a ‘Jacksonian’ sequence, beginning with the brainstem and proceeding through thalamus, basal ganglia, white matter and cortex.

### Brainstem dysfunction

As described in the Introduction section, the brainstem plays a fundamental role in providing the arousal substrate for consciousness. The classical ARAS, originally described by Moruzzi and Magoun^16^ and refined through systematic lesion studies,^21,22^ provides a framework for understanding how several rare pathological processes produce coma.

Rare fungal infections, for example, demonstrate how direct compromise to established brainstem consciousness circuits rapidly precipitates unconsciousness. Central nervous system infection by *Cladophialophora bantiana* targets the ARAS through both direct brainstem lesions and secondary compression, with pontine abscesses producing coma and absent vestibulo-ocular reflexes consistent with classical brainstem models of consciousness disruption; in one reported case, the subacute course culminated in rapid neurological decline despite multi-agent antifungal therapy, with no recovery of arousal.^115^  *Aspergillus* periaqueductal brainstem abscesses likewise can cause coma through direct impairment of brainstem arousal centres and, when intracranial pressure becomes refractory, may progress to brainstem compression and brain death.^116^ By contrast, *Cryptococcus* infections can cause secondary brainstem dysfunction through herniation and hydrocephalus, with rapid deterioration to coma within days and death within weeks when decompression fails.^117^ Even supratentorial fungal lesions can precipitate coma by exerting mass effect and midline shift,^118,119^ leading to rotational distortion and mechanical compression of the upper brainstem^120^; clinical trajectories in these cases are mixed—some patients improve after repeat drainage and antifungal therapy, whereas others show no meaningful neurologic recovery and die after treatment withdrawal.^118,119^

Inflammatory vascular disorders affecting the upper brainstem provide additional evidence for its criticality in sustaining consciousness. Susac syndrome, a rare autoimmune condition affecting the brain's small blood vessels, can cause extensive midbrain and pontine infarcts leading to coma, respiratory failure and brainstem reflex loss, with fulminant cases progressing rapidly to death within weeks to months.^90,91^ Likewise, primary CNS vasculitis produces multi-territorial infarcts preferentially affecting consciousness when brainstem structures are compromised, particularly in rapidly progressive forms where bilateral large-vessel involvement of the basilar artery and posterior circulation leads to severe disability or death.^101^ Immune checkpoint inhibitor-related CNS vasculitis similarly demonstrates the brainstem's vulnerability, with cases showing basilar artery and posterior cerebral artery involvement resulting in coma and respiratory failure, often with poor outcomes despite immunosuppression.^102,103^

Bickerstaff brainstem encephalitis (BBE), a rare autoimmune disorder, provides further evidence for the role of upper brainstem structures in maintaining consciousness. BBE is defined by a clinical triad of ophthalmoplegia, ataxia and altered consciousness, with comatose states directly linked to the dysfunction of upper brainstem arousal circuits.^78,79^ Neuroimaging in comatose BBE patients can yield variable results, underscoring the condition's complexity. For instance, some patients may present in a deep coma with entirely normal structural MRI scans in early BBE diagnosis, suggesting a reversible, non-structural lesion that can resolve with neurological recovery.^80^ In contrast, other cases, particularly those with fulminant and fatal outcomes, can show extensive bilateral T_2_-weighted hyperintensities throughout the midbrain, pons and medulla.^81^ Autopsy studies offer more direct pathological evidence of brainstem inflammation.^78,79^ While BBE pathology is centred in the brainstem, imaging has shown that the inflammatory process can also involve other mesocircuit structures, such as the thalamus.^79^ Nevertheless, the consistent and severe brainstem pathology across cases reinforces the indispensable role of these structures in regulating arousal. The clinical outcomes in BBE-related coma are highly divergent, ranging from complete recovery to death, likely reflecting the severity and reversibility of the autoimmune-mediated brainstem inflammation.^78,80,81^

Finally, rare cases of drug-induced functional suppression of brainstem consciousness circuits can also precipitate profound coma. Tricyclic antidepressant overdoses produce syndromes virtually indistinguishable from brainstem death—with absent brainstem reflexes, fixed pupils and deep coma, yet complete neurological recovery follows drug clearance.^121,122^ The specificity of brainstem involvement is demonstrated by the complete loss of brainstem reflexes including pupillary, corneal, oculocephalic and vestibulo-ocular reflexes, with some cases showing no spontaneous respiration requiring mechanical ventilation.^121-124^ Critically, these cases present with normal brain imaging, confirming that coma results from functional brainstem suppression rather than structural damage.^122,124-126^

### Thalamic dysfunction

As reviewed in the Introduction section, the thalamus serves as the critical integration hub of consciousness networks, with damage to specific nuclei—particularly anterior and posterior intralaminar and paralaminar regions—producing profound alterations in arousal and awareness.^23^ This central role, established through common coma aetiology studies, is further validated by the pathophysiology of several rare conditions.

Wernicke's encephalopathy, an uncommon but well-recognized coma aetiology caused by thiamine deficiency,^9^ consistently targets the same thalamic regions in cases presenting with coma: bilateral medial thalamic hyperintensities appear in comatose patients, whereas non-comatose patients show only periaqueductal changes with thalamic sparing,^75-77^ though other mesocircuit nodes including striatum and cortex are also typically implicated in coma cases.^75-77^ The clinical progression and outcomes of Wernicke's encephalopathy vary dramatically depending on early recognition and thiamine treatment. When treated promptly with intravenous thiamine, patients can show rapid clinical improvement within days to weeks, with corresponding resolution of MRI abnormalities.^75,76^ However, when cortical involvement occurs alongside thalamic involvement—particularly in the frontal and parietal regions—the prognosis is significantly worse, with such patients often progressing to persistent VS/UWS or death.^75^

Reye syndrome, a rare condition that results in hyperammonemia, likewise shows thalamic targeting with characteristic disease progression patterns. One comatose case revealed restricted water diffusion specifically in central thalamus and midbrain despite normal conventional sequences, suggesting early cytotoxic processes affecting cellular integrity rather than structural destruction.^82^ Even when brainstem structures remain visibly intact, thalamic involvement occurs consistently in coma cases.^83^ The clinical course of Reye syndrome in reported coma cases skews towards acute, reversible encephalopathy with full recovery when oedema and most MRI abnormalities resolve.^82,84,134^ In contrast, severe cases with diffuse cortical laminar necrosis presenting alongside thalamic damage can progress to a chronic DOC.^83^

Human African trypanosomiasis presents another example of thalamic targeting with distinct progression patterns. This rare parasitic disease transmitted by tsetse flies consistently targets the thalamus,^94-96^ though it can affect several mesocircuit components. The disease progresses through characteristic stages, with early periaqueductal involvement in non-comatose patients and bilateral medial thalamic changes in those who develop coma. When diagnosed early and treated with appropriate anti-trypanosomal therapy, patients can show remarkable recovery with resolution of both clinical symptoms and imaging abnormalities within months.^94,95^ However, untreated cases invariably progress to death, and even treated patients may develop chronic neurological sequelae including cognitive impairment.^94^

Overall, these rare conditions provide additional evidence for the conclusion, drawn from common coma aetiologies, that severe central thalamic damage alone may be sufficient to induce acute unconsciousness, while also demonstrating how disease progression and outcomes correlate with the extent and reversibility of injury to thalamus and other mesocircuit structures, particularly the cortex.

### Basal ganglia circuits

As reviewed in the Introduction section, the basal ganglia serve as critical modulators of interactions between mesocircuit nodes, with the mesocircuit hypothesis specifically emphasizing the role of striatal medium spiny neurons and the GPi in supporting anterior forebrain function through their influence on central thalamic activity.^42^ More recent work, however, has also highlighted the potential importance of the GPe in modulating cortico–subcortical dynamics relevant to consciousness.^41^

In support of this framework, case reports of heroin-induced coma offer instructive evidence implicating the globus pallidus, and perhaps the GPe in particular, in impaired consciousness. Neuroimaging in heroin overdose comas frequently reveals bilateral hypodensities in the globi pallidi alongside preserved brainstem function, reflecting the selective vulnerability of these metabolically active nuclei to hypoxic or toxic injury.^85,86^ While these reports do not distinguish between the internal and external segments of the pallidum—a limitation for mechanistic interpretation—the consistent involvement of the globus pallidus highlights its central role within the cortico-basal ganglia-thalamocortical loops that support consciousness. Notably, however, a recent post-mortem analysis of fatal heroin overdose cases found reduced GABAergic dendritic density in the GPe alongside increased GABAergic dendritic density in the GPi.^135^ One possible though speculative explanation for this finding is decreased striatum-to-GPe coupling and increased striatum-to-GPi coupling, which would align with the hypothesis that the GPe and the indirect basal ganglia pathway may play a particularly important role in modulating consciousness (see the Introduction section). Heroin-associated spongiform leukoencephalopathy further implicates the pallidum, with comatose cases showing a range of pathologies: isolated pallidal hypodensities with periventricular white matter changes,^86^ diffuse white matter hyperintensities including pallidal involvement^87^ and grey–white matter junction microhemorrhages targeting the pallidum.^88^ Despite anatomical variability, the recurrent involvement of the globus pallidus across heroin-related coma cases underscores its critical role in sustaining consciousness, with emerging data pointing specifically to GPe-related changes as potentially central to the pathophysiology of these states.

This basal ganglia vulnerability also extends to rare parasitic causes of coma. In human African trypanosomiasis, diverse patterns of cortical, brainstem, white matter and subcortical injury have been reported in comatose patients,^94-98^ but basal ganglia injury—particularly of the striatum—is especially common. Involvement of the posterior putamen,^95^ caudate nuclei^96^ and occasionally the globus pallidus^94^ is reported in comatose presentations, though again without segmental distinction, limiting mechanistic insights. Similarly, while Susac syndrome is best known for targeting deep white matter structures (see below), extensive basal ganglia involvement, including striatum and pallidum, has been documented in comatose cases.^90,91^

The relationship between basal ganglia dysfunction and consciousness is complicated, however, by consideration of degenerative diseases targeting the basal ganglia. Parkinson's disease (PD), for example, involves progressive degeneration of the substantia nigra pars compacta and striatal dopamine depletion—disrupting the very cortico-basal ganglia-thalamocortical loops emphasized in mesocircuit models—yet consciousness is typically preserved throughout disease progression. Nonetheless, rare cases of PD-related prolonged unresponsiveness or akinetic coma have been reported.^130-133^ These episodes, sometimes triggered by missed medications but often occurring spontaneously, may last from minutes to over 24 h and resolve fully. Their transient, reversible nature suggests a temporary circuit-level collapse within dopamine-dependent basal ganglia–thalamic networks.

The preservation of consciousness in typical PD, however, poses an important interpretive challenge given the anatomical frameworks reviewed here. We speculate that the preservation of consciousness in typical PD may be explained by both the specific pathways affected and the relative sparing of key mesocircuit nodes. While PD leads to dopamine depletion that preferentially disrupts the direct basal ganglia pathway, the indirect pathway connecting the striatum and GPe (which may be especially important for consciousness maintenance—see above) appears to remain relatively intact structurally and functionally.^136^ Additionally, longitudinal neuroimaging studies demonstrate that while PD causes widespread cortical atrophy, two critical mesocircuit components—the thalamus and globus pallidus—show remarkable preservation even in moderate-to-severe disease stages.^137^ This preservation, particularly of the indirect striato-GPe pathway, may explain why consciousness typically remains intact despite significant striatal dysfunction and cortical degeneration in PD. Indeed, in most of the aforementioned (non-PD) acute coma cases, basal ganglia damage occurred alongside injury to other mesocircuit components—such as the thalamus, brainstem or extensive white matter pathways—rather than in isolation. This pattern suggests that basal ganglia dysfunction contributes to—but is not sufficient for—coma induction. The preserved consciousness in most PD patients, despite profound basal ganglia pathology, supports this view and implies that these circuits may play a modulatory rather than indispensable role in maintaining consciousness. The temporal dynamics of disease progression may be equally important in explaining preserved consciousness in PD. The typically slow progression over years to decades likely allows for compensatory mechanisms that are unavailable in acute lesions affecting the same structures. This compensation may involve functional reorganization within remaining circuits, trophic support from preserved structures or upregulation of alternative neurotransmitter systems. Future experimental studies—such as circuit-specific manipulations in animal models or high-resolution imaging of GPe function in patients—will be essential to test these hypotheses.

### White matter pathways

Common causes of coma also consistently disrupt the deep ‘bridging’ interior zone—a well-defined white matter compartment intercalated among subcortical structures and the ventricular system.^138^ This compartment contains commissural fibres, projection systems (internal capsule and corona radiata) and limbic tracts. Of particular relevance to consciousness, the internal capsule contains functionally distinct fibre bundles including thalamocortical projections from central thalamic nuclei, corticothalamic feedback pathways, thalamostriatal connections from intralaminar nuclei to the striatum and direct projections from brainstem reticular nuclei to cortex.^139-143^ These specific pathways are anatomically intermingled with motor, sensory and other projection systems within the internal capsule, creating a vulnerability wherein damage to this region can simultaneously disrupt multiple consciousness-critical circuits. Such disconnection also provides a straightforward substrate for potential diaschisis effects, with potential remote functional depression in intact cortex, thalamus or striatum secondary to tract interruption.

The importance of these white matter pathways in sustaining consciousness has been established through studies of common coma aetiologies. Diffusion tensor imaging studies in traumatic diffuse axonal injury, for example, show reduced fractional anisotropy in the deep bridging zone, reflecting disrupted cortico-cortical, corticostriatal, thalamostriatal and thalamocortical communication.^144,145^ Similarly, in post-anoxic coma, early CT imaging reveals loss of grey–white distinction in the internal capsule as a robust predictor of poor outcome.^146^ Yet, a critical interpretive challenge remains in both these well-studied aetiologies and the more rare aetiologies reviewed below: even when internal capsule injury is clearly visible, it is difficult to distinguish whether consciousness loss arises from disruption of specific mesocircuit pathways versus the cumulative effect of widespread white matter damage, or collateral injury to directly adjacent thalamic or pallidal regions, which may be poorly delineated in some case reports. Moreover, damage to motor thalamocortical fibres that traverse the internal capsule can result in CMD, which might be mischaracterized as unconsciousness in patients who are in fact covertly aware.^147^ These interpretative challenges have important implications for understanding consciousness mechanisms and developing targeted therapeutic interventions in both common and rare coma aetiologies.

Heroin-associated encephalopathies exemplify this interpretive difficulty. One report described two overdose comas—one with bilateral pallidal lesions plus internal-capsule hypodensities, another with focal periventricular white-matter injury—despite intact brainstem reflexes.^85^ In another report of heroin-induced ‘chasing-the-dragon’ leukoencephalopathy, bilateral pallidal and thalamic lesions accompanied diffuse periventricular white matter abnormalities and corpus callosum involvement in coma cases, while non-comatose patients showed only limited white matter changes.^86^ Other reports implicate extensive damage to the deep bridging compartment in heroin-associated coma: Bega *et al*.^88^ documented extensive diffusion restriction throughout this deep bridging zone in an acute heroin-associated leukoencephalopathy patient presenting with coma, and Rizzuto *et al*.^87^ described diffuse vacuolation of internal capsule myelin lamellae in a delayed spongiform leukoencephalopathy case that progressed to coma. Similar deep white matter damage has been reported in cocaine-induced coma.^148^ While these findings suggest that deep bridging zone damage correlates with consciousness loss, the extensive nature of the pathology in these cases makes it difficult to determine whether consciousness loss results from disruption of specific mesocircuit pathways versus the sheer volume of damaged connections. Moreover, these patterns of deep white matter involvement, particularly affecting the internal capsule, raise the possibility that some patients may experience CMD rather than true unconsciousness.

The complexity of distinguishing pathway-specific versus volume-dependent effects, and inferring unconsciousness from unresponsiveness, is further illustrated by autoimmune and demyelinating disorders. Multiple sclerosis (MS), which characteristically produces periventricular white matter lesions, rarely results in coma, but visual masking studies demonstrate delayed access to consciousness in MS patients,^149-151^ suggesting that even modest deep bridging zone damage can affect the contents of awareness. However, exceptional cases suggest that MS can result in coma when lesion burden reaches critical thresholds within deep white matter pathways. Yetimalar *et al*.^89^ reported two MS patients whose initial presentation was coma (both of whom achieved full recovery), accompanied by bilateral involvement of periventricular white matter, corpus callosum and pons, though the severity of white matter injury compared to non-comatose presentations was not quantified, leaving the threshold for consciousness loss unclear. The involvement of the pons alone does not explain the comatose presentation, as pontine involvement is typical in MS, affecting cerebellar rather than arousal pathways.^152^ Rather, coma in MS may reflect severity or bilaterality of deep bridging zone damage: most MS cases show characteristically small, patchy, perivenous lesions that typically preserve surrounding white matter tract integrity,^153^ internal capsule axonal damage is often asymmetric and modest in severity,^154^ and neuropathological studies indicate that although internal capsule lesions are common in MS, extensive axonal loss remains rare.^155^ Thus, the rarity of coma in MS, despite frequent involvement of consciousness-relevant brain regions, suggests that very extensive damage to specific pathway bottlenecks within the deep bridging zone is required to produce unconsciousness. This interpretation gains support from cases of coma from anti-NMDA receptor encephalitis, a rare autoimmune condition in which antibodies target NMDA receptors, where the degree of internal capsule structural injury (quantified by diffusion metrics) correlates significantly with consciousness impairment severity,^156^ providing more direct evidence for a dose-dependent relationship between deep bridging zone white matter damage and consciousness level. However, as noted above, the internal capsule is directly adjacent to other key mesocircuit nodes, necessitating cautious interpretation of these findings.

Several other rare coma aetiologies similarly demonstrate deep white matter involvement, though again the specificity of pathway involvement remains unclear. In addition to the brainstem damage noted above, fulminant Susac syndrome produces characteristic ‘snowball’ corpus callosum infarctions and ‘string of pearls' internal capsule lesions^90-93^; human African trypanosomiasis creates confluent hyperintense lesions throughout corona radiata, internal capsules and periventricular white matter^94-98^; and thrombotic thrombocytopenic purpura, a rare blood disorder causing microscopic clots, can show periventricular white matter impairment with T2 hyperintensities at ventricular poles in comatose presentations.^99^ Similarly, though coma resulting from fungal infections likely more typically acts through upper brainstem damage (see above), it can also involve deep bridging zones with corona radiata^115^ and periventricular white matter lesions.^116^

This convergence of autoimmune demyelination, toxic-metabolic injury, microangiopathy, fungal infection and parasitic penetration on similar white matter regions provides compelling, though circumstantial, support for the importance of specific connections in sustaining consciousness. As a point of contrast, the preservation of consciousness in corpus callosotomy despite extensive interhemispheric disconnection^157^ suggests that specific thalamocortical, corticothalamic, thalamostriatal, corticostriatal or reticulocortical pathway disruption, rather than deep white matter damage *per se*, likely precipitates acute unconsciousness in the coma aetiologies reviewed herein. Future studies using advanced tractography to map specific pathway involvement in comatose versus conscious patients will be essential to determine whether consciousness loss results from damage to particular mesocircuit connections or simply reflects the cumulative burden of white matter disruption. Such studies should employ standardized consciousness assessments and quantitative tractography methods across multiple rare aetiologies to establish definitive pathway-consciousness relationships.

### Cortical disruption in coma

While brainstem structures provide the foundational substrate for arousal,^21,22^ widespread cortical integrity is equally critical for sustaining awareness, or the contents of conscious experience.^158^ The mesocircuit hypothesis proposes that impaired consciousness results from disruption of anterior forebrain systems, including the striatum, thalamus and broadly defined frontoparietal cortex.^42^ However, the precise cortical regions necessary for conscious awareness remain actively debated, and the relationship between structural cortical damage and functional impairment in consciousness is complex and incompletely understood. Growing evidence supports a posterior cortical ‘hot zone’ encompassing temporal, parietal and occipital regions as critical for the contents of experience.^158,159^ Advocates of this view acknowledge that frontal areas may subserve attention, cognitive control or task reporting rather than consciousness itself,^159^ while others suggest that methodological limitations may have led to underestimations of prefrontal involvement.^160,161^ This ongoing theoretical debate about cortical localization highlights the need for careful interpretation of rare case findings, as isolated reports may reflect reporting biases or task-specific functions rather than core consciousness requirements. Within this evolving framework, rare vascular and metabolic causes of coma offer key insights: they suggest that the extent and severity of cortical network disruption may be more predictive of consciousness level than simple regional localization.

MELAS (Mitochondrial Encephalopathy, Lactic Acidosis, and Stroke-like episodes) syndrome, a rare genetic disorder, provides compelling examples of the complex relationship between cortical involvement and consciousness level. However, individual cases show considerable variability that challenges both simple regional specificity and pure extent-based models: in one case, limited right occipital involvement produced only transient visual field defects with full recovery, while in another case, initial left temporo-parieto-occipital involvement progressing to widespread cortical damage produced refractory seizures, deep coma and death.^104^ Notably, subcortical structures including striatum and thalamus remained relatively spared in the coma case, demonstrating that extensive cortical dysfunction can abolish consciousness even when other mesocircuit nodes remain structurally preserved, though functional impairment through diaschisis may also affect these apparently intact regions.

Severe hypoglycaemia provides another metabolic lesion model that primarily foregrounds cortex: while striatal involvement is frequently reported, neuropathology and MRI show that coma can arise with prominent neocortical laminar injury alongside relative sparing of thalamus and brainstem.^107-110^ PET imaging potentially points to posterior ‘hot zone’^158,159^ dysfunction rather than global cortical dysfunction as the driver of hypoglycaemic coma, with depressed metabolism observed in parietal, temporal and occipital lobes, and recovery of consciousness coinciding with normalization of metabolism in occipital and parietal lobes.^111^ Moreover, though there are focal diffusion changes in deep white matter bridging zones in hypoglycaemic encephalopathy, these largely revert in patients who recover after acute coma; however, more diffuse bilateral hemispheric bridging-zone injury tracks poor recovery (e.g. progression to VS/UWS) when paired with extensive cortical or basal ganglia damage.^112,113^ Indeed, in severe and prolonged hypoglycaemic encephalopathy, structural imaging is abnormal in the majority of patients, with cortical atrophy common and diffusion tensor imaging consistently showing widespread reductions in deep bridging zone fractional anisotropy.^114^ These results suggest that although cortical dysfunction may be the primary driver of hypoglycaemic coma, deep bridging zone damage may partly determine patient recovery outcomes.

The possibility that diffuse cortical network disruption rather than specific regional involvement determines coma severity receives broader support from primary CNS vasculitis, where comatose patients show widespread bilateral infarcts spanning multiple vascular territories.^101^ Compared to focal abnormalities in non-comatose cases, diffuse lesions in comatose CNS vasculitis patients appear in frontal, temporal, parietal and occipital cortices.^102,103^ The simultaneous involvement of both anterior and posterior cortical regions makes adjudicating between competing consciousness theoretical frameworks difficult, as these cases suggest awareness may depend on distributed functional cortex networks rather than localized consciousness centres.

Widespread cortical network disruption, rather than focal damage, may also underlie acute coma in several rare aetiologies through mechanisms beyond direct structural injury. In thrombotic thrombocytopenic purpura, a rare blood disorder in which clots form throughout the body, patients may develop deep coma despite normal brain imaging, likely due to diffuse endothelial dysfunction and microvascular occlusion impairing widespread cortical perfusion; prompt plasma exchange enables full recovery in most cases, though untreated patients typically die.^162^ Functional imaging supports this interpretation, with comatose patients showing bilateral cerebral hypoperfusion, while conscious patients exhibit only focal parietal deficits,^163^ illustrating how consciousness can be disrupted through functional impairment in the absence of visible structural damage (perhaps through diaschisis effects). Similarly, acute intermittent porphyria, a rare genetic disorder affecting heme synthesis, can lead to temporary coma through symmetric cortical lesions spanning frontal, parietal and occipital lobes..^128^ Finally, cerebral venous sinus thrombosis is associated with coma when posterior cortical oedema and diffuse venous congestion are present, in contrast to localized thrombosis in conscious patients, with most patients recovering fully when treated promptly.^129^

These disruption patterns demonstrate that consciousness can be lost through diverse mechanisms affecting diffuse cortical function, rather than solely through damage to specific anatomical regions. However, caution is warranted in interpreting these findings, as they are often derived from isolated case studies with limited generalizability. Broader, systematic studies are needed to clarify the extent to which diffuse cortical dysfunction alone versus posterior dysfunction or frontoparietal dysfunction can reliably predict consciousness outcomes across these rare aetiologies.

## Rare and overlooked causes of prolonged DOC

Unlike acute coma, which often reflects transient network disruption with substantial potential for recovery, prolonged or chronic DOC are typically associated with more enduring structural disconnection across cortical and subcortical systems.^3,25,164^ Whereas coma involves suppression of both arousal and awareness—often due to disruption of brainstem or diencephalic arousal systems—patients in prolonged VS/UWS or MCS exhibit arousal, such as spontaneous eye-opening and sleep–wake cycling. This pattern suggests partial or complete functioning of the upper brainstem, even in the absence of awareness.

Here, we review cases encompassing two distinct trajectories: (i) we primarily focus on progressive neurodegenerative conditions that lead to unresponsive states through gradual deterioration of awareness networks while preserving arousal, which represents a fundamentally different pathway to unconsciousness than acute injury, and (ii) where instructive, we also discuss rare conditions where progression from acute insult to chronic VS/UWS or MCS represents a typical outcome. As we show, these rare or overlooked aetiologies of prolonged DOC demonstrate how diverse pathophysiological mechanisms converge on key anatomical substrates emphasized by the mesocircuit hypothesis (Fig. 1, Table 2).

**Table 2:** A summary of rare and/or overlooked aetiologies with documented cases of prolonged DOC, including both progressive degenerative conditions that lead to unresponsiveness without initial coma and cases where chronic VS/UWS represents a typical outcome following acute injury

Aetiology	Nature of condition	Primary sites affected	References
Adrenoleukodystrophy	Genetic peroxisomal disorder	Periventricular white matter, internal capsule, corpus callosum, pons	^165-169^
Osmotic demyelination syndrome	Metabolic (electrolyte disturbance)	External capsule, thalamus, cortex, basal ganglia, pons	^170,171^
Progressive multifocal leukoencephalopathy	Viral demyelination (JC virus)	White matter (internal capsule, corpus callosum), basal ganglia	^172,173^
*Baylisascaris procyonis* infection	Parasite	Periventricular white matter	^174^
Neuronal ceroid lipofuscinosis	Lysosomal storage disorder	Cortex, thalamus, basal ganglia (striatum, pallidum), periventricular white matter	^175-182^
Progressive supranuclear palsy	Neurodegenerative tauopathy	Thalamus, midbrain, striatum, cortex	^183^
Cerebral amyloid angiopathy	Protein aggregation (vascular amyloid, tau, Lewy pathology)	Periventricular white matter, corona radiata, brainstem, basal ganglia, thalamus, cortex	^184,185^
Anti-NMDA receptor encephalitis	Autoimmune encephalitis	Cortex (frontal/temporal), basal ganglia, white matter	^186-188^
Huntington's disease	Genetic neurodegeneration	Striatum (caudate)	^189,190^
Dementia (e.g. Alzheimer's disease)	Progressive neurodegeneration	Cortex (frontal, temporoparietal), with preserved brainstem	^189,191-193^
Creutzfeldt–Jakob disease	Rapid neurodegeneration	Cortex	^194-197^
Carbon monoxide poisoning	Toxic hypoxic–ischemic injury	Cortex, globus pallidus	^198^
Subacute sclerosing panencephalitis	Viral infection (measles virus)	Diffuse cortical network (posterior-to-anterior spread), basal ganglia (in a minority), periventricular white matter	^199-203^

### Thalamic dysfunction

As reviewed in the Introduction section and our discussion of rare causes of acute coma, the thalamus is the central hub of both arousal and awareness networks. As we review here, this role is likewise apparent in several rare causes of prolonged DOC.

Neuronal ceroid lipofuscinosis (NCL), a group of inherited lysosomal storage disorders, provides perhaps the clearest demonstration of this thalamic vulnerability through its invariable progression to VS/UWS across multiple disease variants. The temporal patterns prove particularly revealing that infantile NCL (caused by a mutation in *CLN1*) reaches VS/UWS by 2–5 years with thalamic volume loss already evident by 0.88 years,^175^ and late infantile NCL (caused by a mutation in *CLN2*) progresses to VS/UWS around age 9 with thalamic cytoplasmic inclusions, cell death and hypometabolism.^176-178^ Studies of animal models of infantile NCL reveal that this thalamic pathology follows a characteristic pattern: while the disease predominantly affects sensory relay nuclei, there is early and persistent astrocytic activation within the central thalamus^179^; likewise, human neuroimaging of the juvenile (*CLN3* mutation) form demonstrates specific atrophy in intralaminar nuclei critical for consciousness.^180^ Thus, while the progression to VS/UWS in NCL also involves diffuse cortical dysfunction (see below), the involvement of both sensory relay and central thalamic systems suggests that disruption of thalamic integrity is not merely a correlate but a likely driver of the progressive loss of awareness in these conditions.

The likely role of thalamic dysfunction in driving consciousness loss is also apparent in progressive supranuclear palsy, where converging pathological processes may specifically target central thalamic function. One documented case progressed over 8 years from orthostatic symptoms to VS/UWS without intervening acute coma, with Fluorodeoxyglucose-Positron Emission Tomography revealing profound thalamic and striatal hypometabolism despite preserved gross anatomy at autopsy.^183^ A likely underlying mechanism becomes clear through the patient's reported histopathology: loss of noradrenergic and dopaminergic inputs likely diminished central thalamic and striatal excitability, with reduced striatal output likely disinhibiting the globus pallidus and thus further increasing thalamic inhibition; moreover, cortical tau pathology may have reduced or impaired corticothalamic feedback. This constellation of changes could represent a classic example of diaschisis, where multiple remote but interconnected lesions—affecting brainstem monoaminergic nuclei, striatum and cortical regions—may have produced cascading functional depression throughout the mesocircuit network, ultimately converging on thalamic dysfunction. Though speculative, this explanation demonstrates the potential generalizability of the mesocircuit hypothesis, which emphasizes thalamic hypometabolism as a driver of prolonged DOC, to aetiologies beyond those that formed the basis for this circuit-based framework, and suggests that diaschitic mechanisms may be a common pathway through which diverse pathologies ultimately compromise consciousness. Moreover, the long progression in this patient before the onset of VS/UWS suggests substantial adaptive capacity that may have ultimately failed when cumulative dysfunction across multiple mesocircuit nodes exceeded compensatory limits.

### Basal ganglia circuits

While the basal ganglia appear to play a modulatory role in acute consciousness loss, their contribution to prolonged DOC may be more substantial, particularly when dysfunction develops gradually or occurs in the context of widespread network failure.

Carbon monoxide poisoning illustrates this principle through selective vulnerability patterns: one VS/UWS case showed severe bilateral globus pallidus degeneration alongside widespread cortical injury and a relatively preserved brainstem.^198^ The functional consequences of this injury pattern become clear when considering that pallidal output normally provides critical inhibitory regulation of thalamic activity, and so its destruction may drive mesocircuit dysfunction. Similar disruptions may occur in osmotic demyelination syndrome, where, beyond the white matter changes described below, prolonged DOC cases frequently show symmetric globus pallidus lesions,^170^ though again without segmental specification. Broader studies (including non-DOC cases) suggest particular vulnerability of the basal ganglia compared to other subcortical structures in osmotic demyelination syndrome,^171^ perhaps suggesting a critical role of these structures in cases that do progress to a prolonged DOC.

This contribution of the basal ganglia to consciousness is also apparent in NCL. Juvenile forms (*CLN3* mutation) typically progress from visual failure (ages 4–6) to parkinsonian features in adolescence and then to VS/UWS without preceding acute coma—a pattern suggesting intact arousal systems despite failing awareness networks. Fluorodopa PET studies reveal significant striatal dopaminergic dysfunction with reduced uptake in putamen and caudate,^181^ while autopsy confirms globus pallidus rarefaction, myelinated fibre loss and neuronal degeneration alongside striatal lipopigment accumulation and astrocytosis.^182^ Thus, basal ganglia dysfunction in these disorders may be as critical to the loss of awareness as is the dysfunction in thalamic circuits reviewed above.

The active role of basal ganglia in consciousness regulation is also apparent in anti-NMDA receptor encephalitis, where hypermetabolism in these structures may actively suppress awareness-supporting cortical networks. PET imaging during disease progression reveals hypermetabolism in temporal lobe and basal ganglia, including both the striatum and globus pallidus, alongside posterior cortical hypometabolism.^186^ Though the heterogeneity of cortical effects is difficult to interpret (see below), the pallidal hypermetabolism is consistent with both mesocircuit predictions^42^ and with PET results in more common DOC aetiologies,^204^ suggesting that pathologically increased pallidal activity may actively inhibit consciousness networks even in uncommon DOC aetiologies.

Finally, even in conditions not typically associated with impaired consciousness, severe basal ganglia degeneration can contribute to the development of prolonged DOC. Huntington's disease offers compelling examples. Although most cases preserve consciousness despite extensive striatal degeneration, some terminal-stage patients progress to VS/UWS, marked by severe caudate atrophy and widespread cortical degeneration.^189^ Early-onset cases with massive cytosine-adenine-guanine (CAG) expansions—such as 92 repeats—may also show rapid progression to unresponsiveness.^190^ This is particularly notable given that Huntington's disease primarily targets the indirect basal ganglia pathway,^205-207^ which, as discussed above, may be especially important for sustaining consciousness via its regulation of thalamocortical activity through the external globus pallidus.

Preserved consciousness in most Huntington’s cases may reflect relative sparing of other mesocircuit components—particularly the thalamus and deep white matter tracts. While the disease produces profound atrophy of the caudate and putamen,^208,209^ the thalamus remains relatively preserved, with some studies reporting no significant volume loss^208^ and others noting only modest reductions.^209^ The case involving 92 CAG repeats cited above^190^ suggests that the extent of genetic expansion—and the resulting burden of brain atrophy—may influence whether consciousness is ultimately lost. Still, findings on the relationship between CAG repeat length and cortical atrophy are mixed: some studies report strong correlations, particularly in frontal regions,^208^ while others find no consistent pattern.^210^ However, longitudinal imaging studies show that longer CAG expansions do correlate with white matter degeneration in the internal capsule,^211^ a critical deep bridging zone containing thalamocortical, thalamostriatal, corticothalamic and reticulocortical fibres essential for consciousness. From a diaschisis perspective, this white matter degeneration could be particularly significant, as these tracts represent the physical substrate through which diaschitic dysfunction may propagate across the consciousness mesocircuit.

Together, these findings suggest that DOC in Huntington's disease may arise only when very large CAG expansions lead to widespread degeneration that overwhelms compensatory mechanisms, with basal ganglia dysfunction contributing to but likely insufficient to cause loss of awareness. The typical preservation of consciousness in Huntington's, like in PD, may reflect not just anatomical sparing but also the temporal dynamics of degeneration, allowing for functional compensation through preserved thalamic circuits and alternative pathways. The critical difference between neurodegenerative conditions that preserve versus abolish consciousness may thus depend on both the breadth of anatomical involvement and whether the rate of degeneration permits adaptive reorganization.

The convergence of these diverse pathological processes, including hypoxic–ischemic injury, osmotic stress, genetic neurodegeneration and autoimmune dysfunction on basal ganglia circuits, underscores their important role in consciousness maintenance. However, the precise mechanisms by which basal ganglia dysfunction contributes to consciousness loss remain speculative and will require more detailed anatomical and functional characterization in future studies, particularly in the rare or overlooked aetiologies reviewed herein.

### White matter damage

The role of prolonged disconnection in chronic DOC is most evident in rare diseases that systematically damage the white matter pathways linking mesocircuit nodes. These conditions reveal that consciousness maintenance depends not merely on preserving individual cortical and subcortical nodes but also on maintaining the structural integrity of specific connections between them.

Adrenoleukodystrophy (ALD), a rare genetic peroxisomal disorder causing very long-chain fatty acid accumulation and progressive demyelination, exemplifies this systematic destruction of connectivity. Childhood cerebral ALD progresses from early cognitive symptoms to VS/UWS or death within 3–5 years,^165,166^ while adult-onset ALD shows more variable progression, with 19% of patients developing cerebral demyelination over 10 years and around 8% progressing to VS/UWS.^167^ The demyelinating process systematically targets periventricular white matter, creating characteristic butterfly-shaped lesions in parieto-occipital regions with corpus callosum and internal capsule involvement—specifically affecting the deep white matter bridging compartment containing commissural fibres, projection systems and reticulocortical tracts.^165-168^ Longitudinal studies clarify the temporal sequence underlying this decline. In the childhood cerebral form of the disease, the progression from the first neurological symptoms to VS/UWS is remarkably rapid, occurring over a mean interval of around 2 years. This clinical deterioration corresponds to a specific anatomical progression: MRI can detect initial, often asymptomatic, lesions in critical pathways like the corpus callosum, internal capsule and pyramidal tracts within the brainstem. The subsequent, rapid spread of demyelination into frontoparietal white matter and deeper brainstem structures is the stage associated with the severe deterioration of consciousness.^169^ However, because the disease rapidly compromises multiple systems simultaneously—including commissural, projection, and arousal pathways—it is difficult to attribute the loss of consciousness to the failure of any single tract. Moreover, given the selective vulnerability of motor pathways in ALD, it is possible that some patients in apparent VS/UWS may retain covert awareness consistent with CMD, though systematic assessment for CMD in this population has not been reported to the best of our knowledge.

While ALD demonstrates progressive destruction of conventional mesocircuit pathways, osmotic demyelination syndrome reveals how acute insults can trigger coordinated network collapse through simultaneous damage to multiple nodes—pontine regions, central thalamus, striatum, globus pallidus and cortical areas.^170,171^ Most intriguingly, one osmotic demyelination syndrome patient who progressed to VS/UWS with preserved brainstem reflexes showed white matter damage specifically in the external capsule,^170^ which contains fibres connecting the claustrum to cortex and interconnecting frontal with occipital, parietal and temporal lobes.^212,213^ This unexpected pattern potentially supports hypotheses regarding claustral contributions to consciousness^213,214^ or theories implicating cortical feedback signalling,^215^ suggesting that consciousness may depend on white matter circuits beyond those traditionally emphasized in mesocircuit models. However, as a single case report, this finding requires replication and should be interpreted cautiously. The patient may have had additional, undetected pathology, or the external capsule damage may have also disrupted adjacent consciousness-critical pathways.

Progressive multifocal leukoencephalopathy (PML), caused by the John Cunningham virus, provides unique insights into how selective white matter vulnerability may lead to sustained DOC despite preserved cortical architecture. Among cases progressing to VS/UWS, two showed clear deep bridging zone damage: one developed akinetic mutism and then VS/UWS persisting 11 years alongside progressive bilateral lesions and massive white matter volume loss,^172^ while another VS/UWS case exhibited confluent bilateral hemispheric demyelination including internal capsule involvement.^173^ The preservation of cortical structure in these cases strongly suggests that consciousness loss resulted from disconnection rather than neuronal death, though the extensive nature of the white matter damage again makes it difficult to identify which specific pathways were critical. Other infectious processes leading to prolonged DOC likewise implicate disruption of deep white-matter tracts: infection by the nematode parasite *Baylisascaris procyonis* typically leaves survivors in a persistent VS/UWS, with MRI showing periventricular white matter injury that may progress to global atrophy.^174^ In a case of coma from infection by the parasite *Angiostrongylus cantonensis*, a patient progressed to MCS for 8 months (followed by slow recovery), with MRI showing inflammation along white-fibre tracts, although the precise location or extent of this inflammation was not reported.^216^

Similar disconnection patterns can result from rare vascular pathologies: in familial cerebral amyloid angiopathy, progressive small-vessel disease leads to periventricular white matter disruption, with MRI revealing periventricular hyperintensities with sparing of superficial U-fibres, and post-mortem histology showing rarefaction, pallor and infarcts in corona radiata and periventricular white matter.^184,185^ The selective vulnerability of deep versus superficial white matter in this condition provides additional evidence that consciousness may particularly depend on specific projection pathways contained within the deep bridging zone, rather than on white matter integrity in general.

The convergence of these disparate pathological processes—genetic demyelination, osmotic injury, viral and parasitic infection, and vascular compromise—on similar white matter pathways provides compelling evidence that awareness depends on maintaining specific connections primarily within the deep bridging zone, though the precise identity of these critical pathways remains to be established. Future research using high-resolution tractography and functional connectivity analysis will be essential to move beyond anatomical correlation towards understanding which specific white matter circuits are truly necessary for awareness.

### Cortical disruption

While thalamic dysfunction may serve as a final common pathway for consciousness loss, evidence from both common and rare aetiologies suggests that diffuse cortical dysfunction can also act as a primary driver of prolonged DOC. In many cases, awareness networks deteriorate independently of arousal system failure, with cortical disconnection or degeneration alone perhaps sufficient to impair conscious experience. This pattern highlights the importance of cortical integrity—not just thalamic or brainstem function—in sustaining awareness over time.

For example, cases of anti-NMDA receptor encephalitis progressing to prolonged DOC consistently involve cortical abnormalities, but the relationship between these abnormalities and consciousness outcomes is complex. While anti-NMDA receptor encephalitis frequently begins with acute coma (as noted above), we here examine specific cases progressing to prolonged DOC, which can provide unique insights into the anatomical substrates of sustained unconsciousness. Specifically, one case series reported that one anti-NMDA receptor encephalitis patient with diffuse cortical necrosis and massive supratentorial malacic defects progressed to VS/UWS (and eventually to MCS with bortezomib treatment), while patients who remained partially responsive showed only focal cortical hyperintensities.^187^ However, structural severity alone does not determine prognosis. In one case, a patient with significant frontotemporal atrophy and hypoperfusion during prolonged unresponsiveness eventually achieved near-complete recovery over 5 years.^188^ Metabolic imaging adds further complexity. As noted above, PET scans across anti-NMDA receptor encephalitis patients (including non-DOC cases) reveal coexisting hypermetabolism in frontal and temporal cortices and basal ganglia, alongside hypometabolism in posterior regions such as the precuneus, angular gyrus, postcentral gyrus and posterior cingulum.^186^ These findings resist reductionist regional theories: the posterior hypometabolism aligns with posterior ‘hot zone’ models of consciousness, while the frontal hypermetabolism may reflect aberrant anterior forebrain activity consistent with mesocircuit disruption.^42,159^ Together, these observations suggest that both structural and metabolic changes in anti-NMDA encephalitis affect consciousness in a regionally heterogeneous and dynamically evolving manner.

The puzzle of how cortical dysfunction translates to prolonged unconsciousness finds clearer resolution in NCL, where progressive cortical degeneration follows predictable patterns across disease variants. As noted above, NCL invariably involves pronounced thalamic atrophy. However, the degree of cortical structural damage appears to correlate with progression to VS/UWS. Infantile NCL (*CLN1* mutations) shows logarithmic cerebral volume decline reaching VS/UWS by 2–5 years,^175^ while late infantile NCL exhibits severe bilateral parieto-occipital cortical atrophy with marked cortical hypometabolism.^178^ Late infantile NCL demonstrates extensive frontal cortical neuronal loss with oxidative damage markers and GABAergic interneuron loss.^177^ In striking contrast, in a case of protracted juvenile NCL (*CLN3* mutation), which never progressed to a prolonged DOC, Anzai et al. observed extensive lipopigment deposits in cortical pyramidal neurons but preserved cytoarchitectures including lamination throughout the cerebral cortex, with no severe neuronal loss.^182^ This suggests that consciousness loss in NCL requires gross structural cortical disorganization, not merely metabolic dysfunction or selective neuronal lipopigment accumulation. The invariable progression to VS/UWS in some forms of NCL, despite its gradual onset, contrasts strikingly with the preserved consciousness in other gradual neurodegenerative diseases such as PD. This difference may reflect the broader anatomical involvement in NCL—simultaneously affecting cortex, thalamus and basal ganglia—which likely overwhelms compensatory capacity. In other words, NCL's multisystem degeneration may exceed the brain's adaptive capabilities regardless of temporal dynamics.

Subacute sclerosing panencephalitis, a rare disease caused by measles virus infection, provides another compelling example of how progressive cortical degeneration can drive consciousness loss while preserving arousal mechanisms. Clinical studies demonstrate that cognitive decline and behavioural changes are universally present from disease onset, with subsequent development of structural cerebral atrophy in a majority of cases in later stages of the disease.^199,200^ While subacute sclerosing panencephalitis also involves subcortical structures including basal ganglia in a substantial minority of cases, as well as deep bridging zone white matter changes in a majority of cases, the progression to VS/UWS appears fundamentally driven by diffuse cortical network failure.^199-201^ Indeed, the disease follows a characteristic posterior-to-anterior cortical progression, with this cortical spread directly corresponding to the progression to eventual VS/UWS.^202,203^ This clinical trajectory illustrates how extensive cortical network disorganization can drive prolonged DOC.

This conclusion is further underscored by the neurodegenerative trajectory of Creutzfeldt–Jakob disease, a transmissible spongiform encephalopathy, which, though rare, is a well-recognized cause of DOC.^9^ The disease's progression can invert the order typically seen in severe acute brain injury, with a confused state rapidly leading to VS/UWS, which is then followed by coma.^194^ The disease involves widespread degeneration throughout the cerebral cortex,^195^ which can appear as ‘cortical ribboning’ on MRI scans.^196^ Functional imaging reveals a corresponding pattern of severe hypometabolism, initially affecting the lateral frontal and parietal cortices before spreading to other brain areas, including thalamus and striatum,^197^ which are both key nodes of the consciousness mesocircuit. Neuropathological examination, however, shows relative sparing of these subcortical structures compared to cortex,^195^ suggesting that the primary driver of progression to DOC in Creutzfeldt–Jakob disease is diffuse cortical degeneration, with possible diaschisis effects in other mesocircuit nodes.

This principle—that profound cortical degeneration can directly lead to DOC—is further supported by slower neurodegenerative dementias, where awareness networks progressively deteriorate while brainstem arousal systems often remain intact. The role of dementia in producing prolonged DOC has likely been underrepresented, with one study reporting that 35.2% of long-term care dementia patients met clinical criteria for VS/UWS.^191^ Progressive dementias can culminate in states of profound unresponsiveness, marked by extensive cortical degeneration—particularly in frontal and temporoparietal regions—despite preserved arousal capacity.^192,193^ This trajectory highlights the critical importance of cortical integrity in sustaining awareness over time. The precise anatomical basis of potential loss of awareness in dementia remains unclear, however: Walshe and Leonard noted that the overall degree of cortical atrophy does not reliably distinguish dementia patients diagnosed as vegetative from those who are not,^189^ suggesting that specific patterns of cortical damage may be more relevant than total atrophy burden. Yet, this observation contrasts with findings from other aetiologies reviewed above, where diffuse cortical network dysfunction appears more predictive of prolonged DOC than any specific anatomical distribution.

This apparent inconsistency may reflect a fundamental limitation of behavioural consciousness assessment in dementia. The combined loss of language, praxis and recognition in extreme-stage dementia creates a maximal aphasia-apraxia-agnosia syndrome that abolishes the very behaviours used to index awareness. Clinical consciousness assessments such as the Coma Recovery Scale-Revised^217^ rely on object manipulation for praxis, object recognition for visual processing and language comprehension or production—all profoundly impaired in advanced dementia. Consequently, some patients diagnosed with VS/UWS based on behavioural criteria may retain covert awareness but lack the cognitive and motor capacities required to demonstrate it. This could explain why cortical atrophy extent fails to distinguish between dementia patients diagnosed with VS/UWS and those who are not: the diagnosis may reflect assessment limitations rather than true consciousness level. Further investigation using neurophysiological and neuroimaging approaches that bypass behavioural output requirements will be essential to determine whether anatomical patterns or assessment limitations account for the heterogeneous consciousness outcomes in late-stage dementia.

Amyotrophic lateral sclerosis (ALS) provides an instructive contrast that clarifies this distinction between motor output failure and genuine consciousness loss. ALS primarily causes progressive motor neuron degeneration, which can culminate in locked-in syndrome^218^—a state that can superficially resemble VS/UWS but preserves intact consciousness.^9^ This demonstrates that even complete motor system degeneration, including motor cortical areas, does not impair consciousness *per se* but rather creates an output bottleneck masking intact awareness. However, approximately 14% of ALS patients develop frontotemporal dementia,^219,220^ where degeneration extends beyond motor cortex into frontal and temporal association areas critical for awareness. Recent evidence suggests that patients who have lost all response capacity in advanced ALS patients with frontotemporal dementia may have progressed beyond locked-in syndrome to actual DOC due to the confluence of motor impairment and frontotemporal cortical deterioration.^221^ This situates ALS patients with frontotemporal dementia within the same continuum as dementia, where widespread cortical network failure may genuinely impair awareness, while pure ALS demonstrates that motor system degeneration alone—however severe—leaves consciousness intact. The ambiguity of such ALS cases also reinforces the importance, more generally, of cautiously disentangling a lack of awareness from behavioural unresponsiveness.

### Beyond categorical boundaries: the case of anti-NMDA receptor encephalitis

As noted throughout our review, DOC can defy neat categorization, challenging both linear models of disease progression and region-specific frameworks for understanding consciousness. Anti-NMDA receptor encephalitis is a particularly illustrative case. Patients often present with acute coma, may evolve into prolonged VS/UWS or MCS, and yet can sometimes recover even after years of unresponsiveness.^188^ This trajectory highlights how a single aetiology can traverse multiple points along the consciousness spectrum, underscoring the limits of static diagnostic categories.

Anatomically and metabolically, anti-NMDA receptor encephalitis exemplifies the distributed network disruption that emerges as a central theme throughout our review. Rather than selectively targeting one mesocircuit node, the condition simultaneously involves multiple components: cortical regions range from focal hyperintensities to diffuse necrosis^187^, basal ganglia show hypermetabolism^186^ and white matter pathways demonstrate structural injury correlating with depth of unconsciousness.^156^ This multi-nodal involvement reinforces our conclusion that consciousness depends on the integrated function of distributed networks rather than on any isolated anatomical structure.

The metabolic patterns observed in this condition further reveal complexities that resist simple interpretation. PET studies show paradoxical hypermetabolism in frontal cortex and basal ganglia alongside hypometabolism in posterior regions such as the precuneus and angular gyrus.^186^ On the one hand, this pattern fits key aspects of the mesocircuit hypothesis: basal ganglia hypermetabolism could drive excessive pallidal inhibition of thalamocortical networks, consistent with circuit-level models and PET findings in other DOC aetiologies.^42,204^ On the other hand, the striatal and frontal hypermetabolism could be read as a direct challenge to those same frameworks.

By sitting at the crossroads of acute and chronic forms, involving several regions simultaneously, and exhibiting both lesion-based and functional disturbances, anti-NMDA receptor encephalitis crystallizes the key themes of our review: it confirms that DOC are best conceptualized along continua rather than rigid categories, that distributed networks of key brain areas rather than single loci underpin awareness and that functional dynamics can modulate the impact of structural injury.

### Clinical implications and future directions

The diverse cases reviewed above highlight a striking anatomical convergence. Despite originating from disparate mechanisms—ranging from autoimmune demyelination to mitochondrial failure, toxic exposures, infectious agents and neurodegeneration—these rare or overlooked conditions consistently target a common set of network components: brainstem arousal systems, central thalamic nuclei, basal ganglia, widespread cortical regions and deep white matter ‘bridging zones’, particularly the internal capsule, which forms the major conduit of mesocircuit connections. This convergence across diverse aetiologies points to a consistent pattern that transcends individual case limitations and strengthens our leading theoretical frameworks despite the inherent variability in evidence quality from rare conditions. The repeated involvement of these structures across diverse aetiologies reinforces the view that consciousness is not localized to any single anatomical structure but rather emerges from the dynamic interplay among distributed systems. In particular, the internal capsule and surrounding deep white matter may represent an underrepresented bottleneck for conscious integration—its disruption, whether by microangiopathy, metabolic failure, infection or demyelination, often correlates with impaired arousal and awareness even when cortical or thalamic structures are relatively spared. These findings lend compelling support to circuit-based models such as the mesocircuit hypothesis and emphasize the importance of preserving not only neural nodes but also their structural and functional connectivity. The possibility of diaschisis, wherein a localized brain lesion causes dysfunction in other typically connected brain regions,^45^ provides an additional explanatory layer for these observations, helping account for the occasional mismatch between structural damage and functional impairment across these diverse aetiologies.

The temporal dynamics of lesion development also emerged in our review as a critical dimension of underexplored causes of prolonged unconsciousness. Progressive conditions like PD typically preserve consciousness despite damage to key mesocircuit nodes, which we speculate may be through compensatory mechanisms unavailable in acute injury, including functional reorganization, trophic support from preserved structures and upregulation of alternative neurotransmitter systems. However, this compensation may have limits: progressive conditions with broader anatomical involvement (such as NCL) or faster progression (such as Creutzfeldt–Jakob disease) may eventually overwhelm adaptive capacity. Future research should investigate these temporal dynamics systematically, examining how rates of degeneration, breadth of involvement and compensatory capacity interact to determine whether progressive diseases culminate in DOC.

Our analysis also exposes a fundamental challenge in consciousness research: the marked disconnect between sophisticated theoretical models and the relative poverty of clinical assessment tools. Contemporary theories of consciousness—including the mesocircuit hypothesis,^42^ integrated information theory^222^ and global workspace theory^223^—make specific predictions about neural dynamics, connectivity patterns and information integration that have the potential to finely distinguish between different components and attributes of consciousness. Yet, clinical assessment remains anchored to crude behavioural indicators: the Coma Recovery Scale-Revised^217^ and similar tools, though highly valuable, evaluate motor responses, visual tracking and command following but cannot probe the underlying neural mechanisms or distinguish between competing theoretical accounts. A patient with thalamic dysfunction from NCL, white matter disconnection from ALD or cortical degeneration from Creutzfeldt–Jakob disease may present identically on behavioural assessment despite fundamentally different pathophysiology and potentially different targets for intervention. The potential diaschisis effects we have noted further complicate this picture: remote functional suppression can produce behavioural unresponsiveness even when consciousness-supporting structures remain anatomically intact, creating a mismatch between structural imaging, functional status and clinical presentation that current assessment tools cannot resolve.

These rare and overlooked aetiologies represent a largely untapped resource for advancing consciousness science. While our understanding of unconsciousness has been shaped predominantly by studies of epilepsy, anaesthesia, sleep, TBI, stroke and anoxic injury, the diverse pathological mechanisms reviewed here offer unique opportunities to test and refine theoretical frameworks. This paper represents only an initial attempt to systematically catalogue these conditions and interpret them through the lens of current consciousness theories. Each rare aetiology provides a distinct natural experiment: by expanding our investigative scope beyond common aetiologies, we gain access to a richer set of lesion models that can adjudicate between competing theories.

From a research perspective, this review highlights critical priorities for advancing our understanding of consciousness. Future studies should employ standardized consciousness assessment protocols across these rare conditions, utilize advanced tractography to identify specific critical pathways, conduct longitudinal investigations tracking consciousness changes with anatomical progression and establish multicentre collaborations to overcome the inherent limitations of small sample sizes in rare condition research. Particular attention should be paid to distinguishing pathway-specific effects from cumulative white matter damage, clarifying the differential roles of basal ganglia subregions and determining causal versus correlational relationships between anatomical damage and consciousness loss. Moreover, developing mechanism-specific biomarkers—such as perturbational complexity index^224^ for network integration, diffusion metrics for white matter integrity or metabolic imaging for regional dysfunction—will be essential to bridge the gap between theoretical predictions and clinical assessment. Such multimodal approaches could reveal whether behaviourally similar presentations actually reflect distinct subtypes of consciousness impairment, each requiring tailored therapeutic strategies.

From a translational standpoint, this convergence suggests a dual therapeutic approach to DOC from both common and rare aetiologies. First, as is standard practice, the initiating pathology must be addressed. But, in many cases, patients will remain trapped in pathological unconsciousness due to residual network failure even after the primary insult is mitigated. Recovery in such cases will likely require concurrent strategies to restore function across multiple nodes in the consciousness mesocircuit. Repairing deep white matter architecture—through agents promoting remyelination, oligodendrocyte survival and anti-inflammatory stabilization—may help re-establish thalamocortical, thalamostriatal and corticostriatal communication. Likewise, systemically acting agents such as incretin-based therapies, which simultaneously act on several pathological processes implicated in DOC, offer promise for multi-site network reactivation.^225^ Clinical trials of circuit-level interventions should be designed to account for the heterogeneous aetiologies and variable recovery trajectories observed in DOC patients, with particular attention to biomarkers that can predict treatment responsiveness across different pathological mechanisms. Future therapeutic frameworks must, therefore, integrate disease-specific aetiological treatments with broader circuit-level interventions. This dual approach—one tailored to pathology, the other to network restoration—may be the key to reanimating the distributed, multilayered systems that support conscious awareness.

## Data Availability

Data sharing is not applicable to this article as no new data were created or analysed in this study.
